# Preparation of Polystyrene Microsphere-Templated Porous Monolith for Wastewater Filtration

**DOI:** 10.3390/ma14237165

**Published:** 2021-11-25

**Authors:** Nur Faezah Ibadat, Suryani Saallah, Clarence M. Ongkudon, Mailin Misson

**Affiliations:** Bioprocess Engineering Research Group, Biotechnology Research Institute, Universiti Malaysia Sabah, Jalan UMS, Kota Kinabalu 88400, Sabah, Malaysia; nurfaezahibadat@gmail.com (N.F.I.); suryani@ums.edu.my (S.S.); clarence@ums.edu.my (C.M.O.)

**Keywords:** porous monolith, polymer, template, homogenous pore, water filtration

## Abstract

Porous monoliths prepared using templates are highly sought after for filtration applications due to their good mass transport properties and high permeability. Current templates, however, often lead to the formation of dead-end pores and irregular pore distributions, which reduce the efficiency of the substrate flow across the monolith column. This study focused on the preparation of a microsphere-templated porous monolith for wastewater filtration. The optimal template/monomer ratio (50:50, 60:40, 70:30) was determined, and appropriate template removal techniques were assessed for the formation of homogenous pores. The physicochemical characteristics and pore homogeneity of the monoliths were examined. The 60:40 ratio was determined to result in monoliths with homogeneous pore distributions ranging from 1.9 μm to 2.3 μm. SEM and FTIR investigations revealed that solvent treatment was effective for removing templates from the resulting solid monolith. The water quality assessments revealed reductions in the turbidity and the total number of suspended particles in the tested wastewater of up to 96–99%. The findings of this study provide insightful knowledge regarding the fabrication of monoliths with homogenous pores that are beneficial for wastewater treatment.

## 1. Introduction

The past decade has witnessed the advancement of monoliths as an important technology in diverse applications including separation [1], filtration [2], biomolecule purification [3] and chromatography systems [4]. This is owing to their fascinating characteristics, which include high surface area, chemical stability, large pore volumes, high permeability and low flow resistance, which enable high-speed separation, making them promising for achieving high throughput, resolution, and separation in short run times. Sol–gel, free-radical polymerization and click reactions are among the methods employed for synthesizing monoliths comprising glycidyl methacrylate, porogen, initiator, and cross-linker [5]. There is much to discover regarding the fabrication of monoliths, as evidenced by the increasing number of studies in the reported literature on monolith applications. Interest in monoliths for wastewater treatment has markedly increased over the past decade, driven by their attractive features, which include interconnected pore structures and convective mass transfer. Gel-emulsion-templated polymeric monoliths have been employed for the efficient removal of particulate matter [6]. Chitosan/MOF composite [7] and porous polyurea [8] porous monoliths have also been used as adsorbents to remove pollutants in water. Cellular glassy porous monoliths have also been prepared using sacrificial paraffin spheres as the porogen and resorcinol-formaldehyde (RF) resin as the carbon precursor in order to achieve electrical and electromagnetic properties [9]. Compared to typical macrocellular materials, monoliths possess porosities in the range of 75–85 percent, and have narrow cell size distributions and microporosity, as well as high surface areas.

Monoliths are fabricated with interconnected macropores smaller than 2 nm in diameter and mesopores ranging from 2 nm to 50 nm in size [10]. The pore structure within monoliths formed using a template allows liquid to flow through the monolith at a reasonable pressure. Biodegradable polymers such as polylactide (Sun et al., 2017) and supramolecular aggregates are common templates that are frequently used in monolith fabrication [11]. The desired pore size, porosity, and pore morphology can be obtained by fine-tuning the properties of templates. However, the templates currently in use present several limitations, such as poor heat dissipation, uneven pore size distribution, the wall channel effect, and low mechanical strength, particularly when scaled up [12]. In addition, the formation of dead-end pores and uneven pore distribution due to partial reaction by free radical initiators lead to the formation of pressure inside the monolith, hampering its commercialization potential [12]. Therefore, templates that are able to produce homogenous and well-structured monolith pores are highly sought after.

Nischang (2013) presented a comprehensive review of the morphology, porosity, nanostructure and chromatographic performance of porous polymer monoliths. The most commonly used radically initiated cross-linking polymerization procedures require initiation in a homogeneous liquid polymerization precursor that contains monomers and porogenic solvent. Porogens have been used to synthesize monodisperse polystyrene microspheres (Vlakh et al., 2011). Porogens, however, have not been used as the directing template for the formation of pore structure, leading to monoliths with irregular pores. As a result, substances distributed in monoliths face complex pathways, thus limiting the efficiency of their adsorption and separation. According to Wu et al. (2012), monoliths with homogenous pore structures would have greater efficiency when employed as an HPLC column. Controlling the porous structure and properties of monoliths has been the main direction of research.

Microemulsion-based polymeric microspheres are a good candidate for monolith templates owing to their ability to fine-tune porosity and particle size. Microspheres are small spherical particles, sometimes referred to as microparticles, with particle diameters ranging from 1 to 1000 µm. Shameer and Nishath [13] generated polymer dispersions made of a variety of monomers, including styrene, butyl acrylate, and methyl methacrylate in the presence of a coupling comonomer. Different hybrid morphologies were obtained depending on the reaction conditions and the surfactants employed. A microemulsion is a thermodynamically stable and transparent system that can be prepared by means of several techniques, including solvent evaporation methods, spray drying, the solution-enhanced dispersion method and the hot melt technique. Among these techniques, the most frequently applied method, due to its ease of preparation, is the solvent evaporation method. Monteiro, et al. [14] discovered that microemulsion performs best with polymers such as polycaprolactone and polystyrene. The effects of the chemical precursor and surfactant concentration on the formation of particles were also reported in our previous study [15]. Among the tested polymers (polystyrene, polycaprolactone, polypropylene, polyethylene, and poly (vinyl-alcohol)), polystyrene was found to be the best chemical precursor for the formation of good particle morphologies with sizes ranging from 1.94 µm to 3.45 µm.

Polystyrene microspheres have been used extensively as hard templates for various hollow spherical materials [16] due to their good features, which include low density, high surface area, excellent charge capacity, and high permeability. These properties have made them highly applicable in various industrially important applications in biocatalysis and immobilization systems [13,17]. Until recently, monoliths with ordered structures prepared using microsphere templates had never been reported. Therefore, polystyrene-based microspheres were investigated as monolith templates in this study. It has traditionally been a major challenge to produce monoliths with a well-ordered structure. Uneven pore distributions can reduce the efficiency of monoliths in water filtration systems. Silica monoliths have been prepared via spinodal decomposition as a unique method for producing homogeneous interconnected pore networks, significantly enhancing mass transport [18]. According to Ali [19], during the templating process, a pre-formed template is filled with soft precursor material to form the desired shape before the precursor material is hardened via a chemical or physical process. The template is removed once the material resembles the shape of the template. Zhang, et al. [20] explained that calcination, chemical etching or special treatment can be employed for the removal of hard templates.

Template removal is also a critical step that requires major consideration. Incomplete template removal results in the drawback of poor pore interconnectivity. A facile procedure for template removal requiring moderate temperature and pressure is a technological interest that is being actively pursued by scholars and industrialists. Malik, et al. [21] successfully used high-temperature solvent extraction to produce physically robust nanostructured silica monoliths. However, their procedures required special equipment high pressures and temperatures (Pc and Tc, respectively), and long processing times.

Hence, in the present work, a procedure for preparing porous monoliths using polystyrene-based microsphere templates is reported. The strategy for incorporating the template with monolith monomers at the best ratio was studied. The removal of the template was carried out via solvent treatment and thermal treatment. The homogeneity of the resultant pores following template removal was investigated. Finally, the performance of the fabricated monolith for wastewater filtration to remove particulate matter was assessed.

## 2. Materials and Methods

### 2.1. Chemicals

Polystyrene, ethylene glycol dimethacrylate (EDMA) 98%, glycidyl methacrylate (GMA) 97%, azobisisobutyronitrile (AIBN) and Brij O10 surfactant were all purchased from Sigma Aldrich (Burlington, NJ, USA). Toluene, dimethylformamide (DMF) were purchased from Fisher Chemical (Hampton, NH, USA). Polyethylene glycol (PEG) was purchased from Tokyo Chemical Industries Co. Ltd. (Tokyo, Japan).

### 2.2. Preparation of Polystyrene Microsphere Template

Polystyrene microspheres were synthesized via solvent evaporation method as described in a previous study [15]. Polystyrene polymer at different concentrations (10–40 wt%) was dissolved in DMF solution. Brij O10 surfactant (7%) was added as a stabilizer. The solution mixture was mixed using a magnetic stirrer at a stirring rate of 1500 rpm at 80 °C (Favorit, PLT Scientific Instruments, Selangor, Malaysia) and left for 1 h under continuous stirring to evaporate the solvent. The resulting polymeric microspheres were kept at room temperature until further use as a monolith template.

### 2.3. Preparation of Microsphere-Templated Porous Monoliths

**Figure 1:**
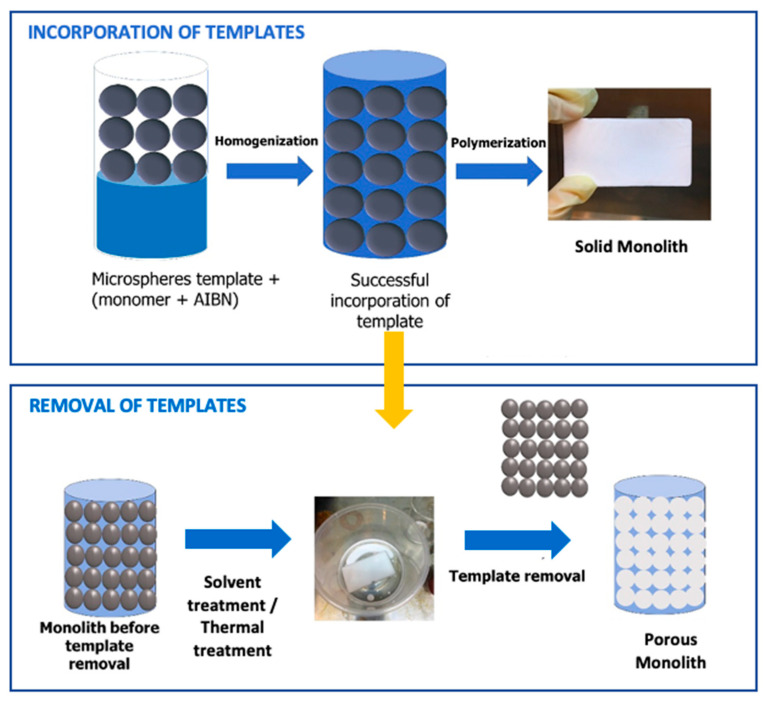
Schematic diagram of the preparation of porous monoliths using polystyrene microsphere particles as a pore-directing template. Part I: incorporation of the template into the monolith monomers. Part II: removal of the template via solvent treatment to produce a porous monolith.

The preparation of porous monoliths using the synthesized microsphere template is schematically represented in Figure 1. The procedure consisted of two parts. Part I: the incorporation of the microsphere template into the monolith monomer. Part II: the removal of the template from the structure of the solid monolith, thus creating pores across the monolith structure.

Hierarchically porous polymethacrylate monoliths (PMMA) were synthesized through free-radical co-polymerization as described by [5] with slight modification (Figure 1; Part I). Glycidyl methacrylate and ethylene glycol dimethacrylate were used as monomers. Polymer solutions containing microspheres were mixed with the cross-linker, functional monomer and AIBN as an initiator to the reaction. Microsphere templates were added into the mixtures at varying template/monomer ratios (50:50, 60:40 and 70:30). The solution was sonicated at 20 °C for 20 min. Subsequently, the solution was inserted into a casting mold and heated at 60 °C for 3 h inside a water bath to allow the polymerization to occur [22]. The resulting solid monolith was removed and kept at room temperature until further use.

Next, the template was removed via solvent treatment and thermal treatment methods (Figure 1; Part II). The solvent treatment was conducted by soaking the monolith in toluene overnight. The monolith was washed with water to remove the solvent residues. Finally, the sample was oven-dried at 60 °C and kept at room temperature until further use [23]. For the thermal treatment method, the monolith was heated at 150 °C for 1 h in an oven. The physical structure of the monolith was observed after the first hour of exposure to high temperatures [24].

### 2.4. Characterization of Microsphere Template and Monoliths

The textural and morphological properties of the polystyrene microsphere templates were observed on a scanning electron microscope (Hitachi High Technologies America Inc. S-3400 Los Angeles, CA, USA) operated at 10 kV. Samples deposited on the sample holder were coated with a conductive gold before morphological examination. Successful incorporation and removal of the microsphere template on the monolith were also confirmed by observing the monoliths using SEM. The average size of the polystyrene microsphere particles at different concentrations was determined using Dynamic Light Scattering (Nanoplus Micromeritics Instrument Corp, Tewkesbury, UK). The average pore size and the pore distribution of the monoliths obtained using different ratios were evaluated using Image J, 1.52 version software on the pre-captured image from SEM observation (Chan et al., [5]). The original SEM image was uploaded and binarized by altering the threshold. Particle size measurements were carried on based on the created particle outline. Fourier transform infrared spectroscopy (Agilents Technology Cary 630, Santa Clara, CA, USA) was used to characterize the functional groups in the monoliths after the incorporation and removal of the templates. The changes were observed by comparing the tested monoliths with a control monolith synthesized without the presence of a template.

### 2.5. Experimental Setup for Wastewater Filtration

**Figure 2:**
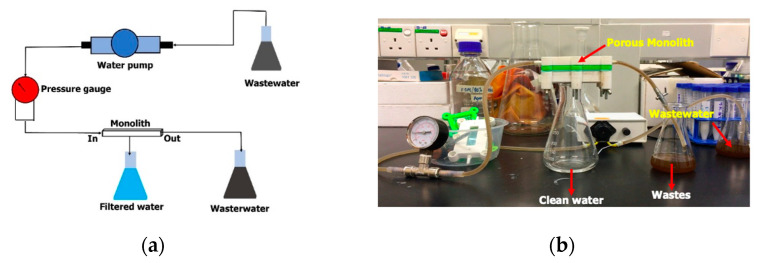
(**a**) Schematic representation of the monolith water filtration system. (**b**) Actual monolith water filtration system using the developed microsphere-templated porous monoliths.

The ability of the resultant porous monoliths to remove particulate matter in wastewater was tested (Figure 2). Wastewater samples showing a high level of turbidity were collected from an animal pond and laboratory waste. The animal pond wastewater was collected from the sea otter pond in Zoo Lok Kawi, Sabah, Malaysia, while the laboratory wastewater was collected from the Pilot Plant Laboratory at the Biotechnology Research Institute, Universiti Malaysia Sabah. A mini pump filtration system was developed (Figure 2a) consisting of a peristaltic pump, pressure gauge, monolith and flasks for the wastewater, clean water and waste residues. About 150 mL of wastewater samples were flowed through the monolith using a peristaltic pump with a pressure lower than 5 kPa. The actual setup of the monolith water filtration system is depicted in Figure 2b. The clean water was collected and tested for turbidity and total suspended solids (TSS) levels.

### 2.6. Water Quality Analysis

Morphological analyses of the samples before and after the filtration process were conducted using an inverted microscope (40× magnification) to qualitatively observe the presence of particulate matter. The turbidity and total suspended solids (TSS) analyses of the samples were performed by an accredited laboratory for water quality surveillance located at the UMS-Water Analysis Laboratory Research Unit, Faculty of Science and Natural Resources, Universiti Malaysia Sabah. The TSS was analyzed using the APHA 2540 D 2012 method, while the turbidity test was carried out using the YSIProDSS method. The pH of the water samples was determined using a pH meter (Cyberscan, Thermo Fisher Scientific, MA, USA).

## 3. Results

### 3.1. Synthesis of Microsphere Particles as Monolith Templates

The average diameter and polydispersity index (PDI) of the polystyrene microspheres at different concentrations of polystyrene (10–40 wt%) are presented in Table 1. It can be seen that the particle diameter increased with increasing polymer concentration. The smallest particles were observed when using 10 wt% of polymer, with an average diameter of 0.9 μm. The particle size was also found to be in agreement with the PDI values. It can be observed that different polymer concentrations produced varying average microsphere diameters. The solution prepared at a lower concentration (10 wt%) produced an average diameter of 0.92 μm, with the lowest PDI value (0.537). According to Danaei, et al. [25], PDI values closer to 0.0 indicate monodisperse solutions, implying a homogenous system, while PDI values closer to 1.0 demonstrate polydisperse solutions, due to the presence of varying sizes of particles. Based on the findings reported by Ibadat, Ongkudon, Saallah and Misson [15], solutions prepared at 10 wt% polystyrene produce well-shaped microsphere particles compared to the formation of aggregated particles at a high concentration (40%). This is in agreement with the findings seen in this study, whereby increasing polymer concentration resulted in the formation of larger particles, ranging from 2.4 to 3.4 μm (Table 1). The experimental results showed that aggregation was more pronounced in the template prepared using a higher polymer concentration, probably as a result of the higher degree of viscosity of the polymer. The non-homogeneous conditions observed at higher concentrations of polystyrene can probably be attributed to the formation of irregular shapes and aggregated particles. This phenomenon was elucidated by Johansen and Schæfer [26], who suggested that polymer concentration strongly influences the viscosity of the solution, leading to particle aggregation.

The findings show that polymer concentration significantly influences the pore distribution and homogeneity of the microsphere particles. With increasing concentration, non-homogenous particles were observed due to the formation of particle aggregation. Polystyrene at 10 wt% was found to be an optimal concentration for good-quality microspheres with a lower PDI value. Hence, this concentration was selected for further study on the preparation of porous monoliths.

### 3.2. Effect of Template/Monomer Ratio on Pore Distribution

**Figure 3:**
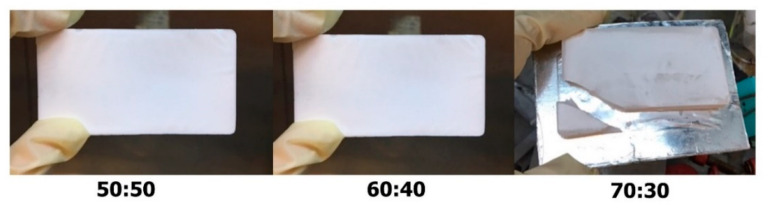
Physical observation of solid monoliths produced using different ratios of templates and monomers (50:50, 60:40, 70:30).

Polymethacrylate monoliths were prepared by mixing cross-linker monomer, functional monomer, pore-directing agent and initiator using free-copolymerization. The synthesized polystyrene microsphere particles were used as templates for the monolith pore-directing agent. Pre-polymerization mixtures have been recognized as a factor influencing the morphology of the resultant monolith [13]. Therefore, the optimal ratio of the template and the monolith monomers (50:50, 60:40 and 70:30) for monolith fabrication was investigated. The physical structures of the resultant monoliths are presented in Figure 3. As can be observed, different monolith/template ratios produced monoliths with different physical structures. While the monoliths produced with the 50:50 and 60:40 ratios presented hard and solid physical structures, the monolith produced with the 70:30 ratio exhibited soft and brittle features.

**Figure 4:**
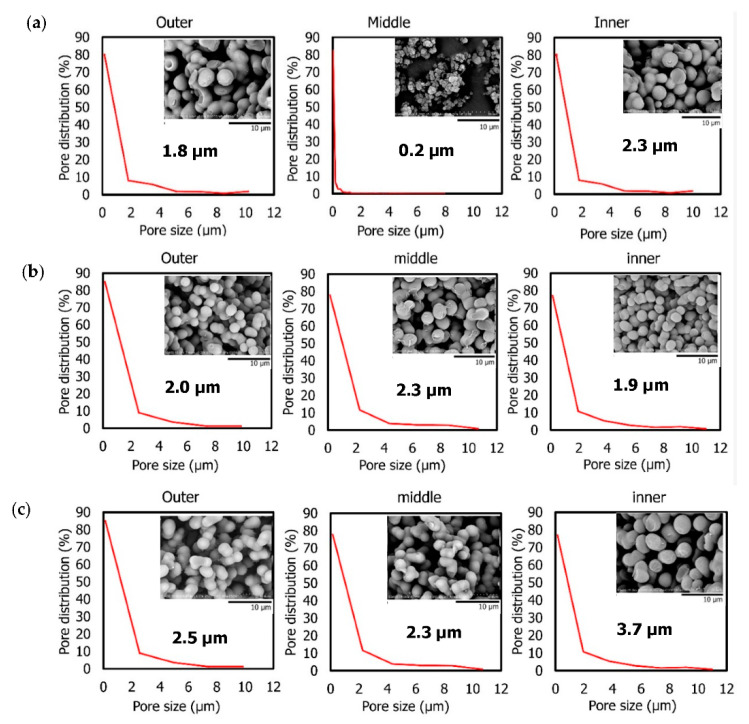
Pore distribution and SEM images (insert) of monolith cross-sections (outer, middle, inner) at different template/monomer ratios: (**a**) 50:50, (**b**) 60:40, (**c**) 70:30.

The internal structures of the three monoliths were further characterized. The pore distribution was observed at the outer, middle and inner monolith cross-sections (Figure 4). The images of each step of the ImageJ analysis are shown in Figures S1–S9. It can be observed from the 50:50 profile section (Figure 4a) that a smaller pore size can be found in the middle section (0.2 μm) of the monolith compared to the outer (1.8 μm) and inner (2.3 μm) sections. The outcomes seen here are in agreement with the image produced by the SEM, whereby smaller and non-interconnected pores were found in the monolith produced with a 50:50 ratio. Interconnectivity among pores is essential, as these connections allow the liquid to flow through the monolith, as well as permitting the occurrence of any sort of diffusion process [27]. The monolith produced with the 60:40 ratio exhibited homogenous particles, with particle diameter ranging from 1.9 μm to 2.3 μm in all sections (Figure 4b). In addition, good particle morphology with well-interconnected properties was observed compared to the particles in the monolith produced with the 50:50 ratio. The monolith produced with a 70:30 ratio demonstrated a lower degree of homogeneity than the 60:40 monolith. Larger particles were generated in the inner section, with sizes of 3.7 µm, followed by the outer (2.5 µm) and middle (2.3 µm) sections. The particles in the outer and middle sections were found to be slightly aggregated.

The physical and internal examinations of the monoliths prepared at various template/monomer ratios indicated the important role played by the template in the production of robust monoliths. The monolith produced with a 70:30 template/monomer ratio had the lowest degree of homogeneity (Figure 4c) and formed a fragile monolith structure (Figure 3). High amounts of template (70%) may result in insufficient reactivity for the building of linking pores throughout the monolith structure, resulting in non-homogeneous pore development, as seen in the SEM images. Some monolith constructions may not be completely covered by monomer components, meaning the structure can be readily shattered.

The ability of the monolith samples to allow water to flow through them was also further investigated. The results reveal that water failed to pass through the monolith with a 50:50 template/monomer ratio (data not shown), owing to the non-interconnected pore features, as shown in Figure 4a. Monolith synthesis using this ratio is not ideal for water filtration systems, since the tiny pore size in the inner section may prevent any substrate from flowing through the surface. According to Geise, et al. [28], simple filtration involves pore flow, whereby separation is predominantly accomplished via a size-sieving mechanism in which the solution is not allowed to flow when the pore size is too small. A similar observation was also found when using the monolith with a 70:30 ratio. Due to its non-homogeneous particles and fragile structure, it was similarly difficult for the water solution to flow through. The 60:40 template/monomer ratio, which exhibited a homogeneous pore distribution for all monolith cross-sections (outer, middle, and inner), was found to efficiently allow water to flow through it. The interconnectivity of the pores may allow liquid solutions to flow from one pore to another, which might be useful in water filtration systems; hence, it was chosen as the basis for future research.

### 3.3. Template Removal from the Monolith Structure

Following their incorporation into the monoliths, the templates are removed, resulting in the formation of pore structures resembling the shape of the templates. The efficiency of solvent treatment and thermal treatment for the removal of the incorporated microsphere templates in monolith structures was assessed and observed through SEM and FTIR analyses.

#### 3.3.1. Morphological Structure Analysis

**Figure 5:**
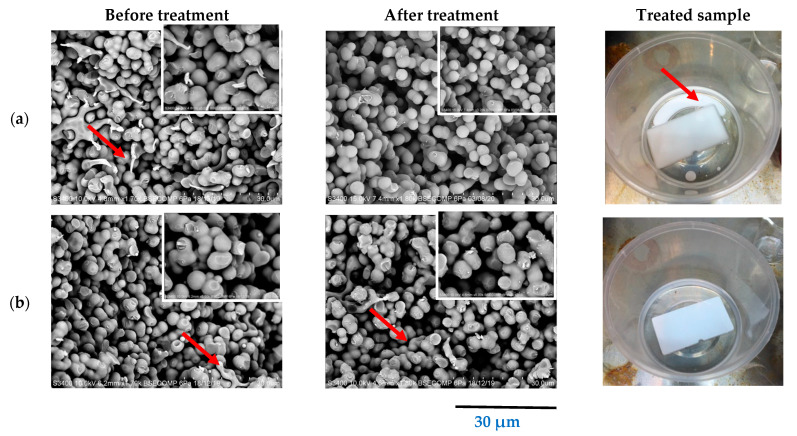
Physical observation and SEM images of monolith before and after template removal process via (**a**) solvent treatment and (**b**) thermal treatment. Red arrows indicate the microsphere template.

Figure 5a,b present the morphological characteristics of the monolith before and after template removal via the solvent treatment and the thermal treatment, respectively. The presence of template can be observed in the SEM images before template removal. After the solvent treatment, template was found to be absent from the images, indicating that the template had been successfully removed from the monolith structure. The monolith exhibited distinct morphologies before and after treatment with respect to the structure of the particles. In addition, a white solution appeared in the solvent, which was probably the microsphere template that had been successfully removed (Figure 5a). Meanwhile, some remaining template was present in the monolith after the thermal treatment (Figure 5b). This indicates that thermal treatment was unable to completely remove the template from the monolith. The findings of this study show that the solvent treatment is superior for the removal of templates from monolith structures. These findings are in agreement with the previous study conducted by Xu, et al. [29], in which it was reported that solvent treatment was superior for polymeric template removal. Solvent treatment has also been reported to be superior for the removal of organic templates from nanostructured silica monoliths, as described by Dabbs, et al. [30]. The resulting monoliths from both the Soxhlet and supercritical extraction methods were found to be mechanically robust, optically clear, and free of cracks. This technique offers the advantage of a moderate temperature and ambient pressure processing without the requirement of specialized equipment.

#### 3.3.2. Chemical Composition Analysis

**Figure 6:**
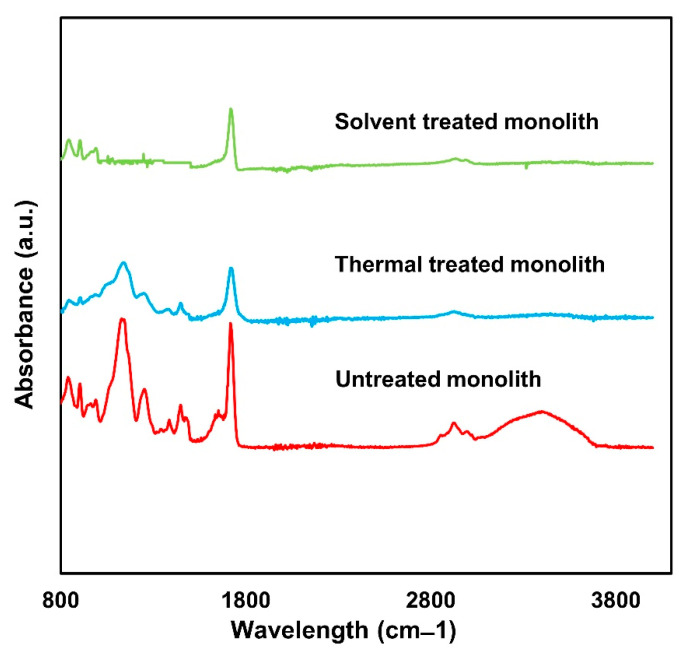
FTIR spectra of untreated monolith, thermal-treated monolith and solvent-treated monolith.

The efficiency of both treatments for template removal was further assessed using FTIR analysis (Figure 6). FTIR identifies the functional groups present in the monolith samples [13]. The absorbance of the samples was tested over a range from 600 cm^−1^ to 4000 cm^−1^. As can be seen from the graph, some peaks disappeared, while some peaks demonstrated a reduced intensity in the solvent-treated monolith. The absorbance peak at 3593 cm^−1^ represents the O-H group of the template surfactant (oleyl alcohol) [31], which disappeared after the treatment process. No obvious peaks were observed within the range 1150–1650 cm^−1^. The peaks at 1155 cm^−1^ and 1386 cm^−1^ represent the C-C and C-H stretching of polystyrene [32], respectively, while the peak at 1255 cm^−1^ corresponds to the C-N amide of dimethylformamide (DMF) [33], which was used as a solvent during the fabrication of the polystyrene template. These observations indicate the absence of the polystyrene template in the solvent-treated monolith, thus implying the complete removal of the template. On the other hand, most of the above-mentioned peaks remained in the monolith after thermal treatment. This observation is in agreement with the presence of template observed in the SEM images (Figure 5b). These findings indicate that the thermal treatment was inefficient for completely removing the template from the monoliths. In conclusion, on the basis of the SEM and FTIR analyses, the solvent treatment was found to be the best technique for the removal of the microsphere template.

### 3.4. Thermal Stability of Microsphere-Templated Porous Monoliths

**Figure 7:**
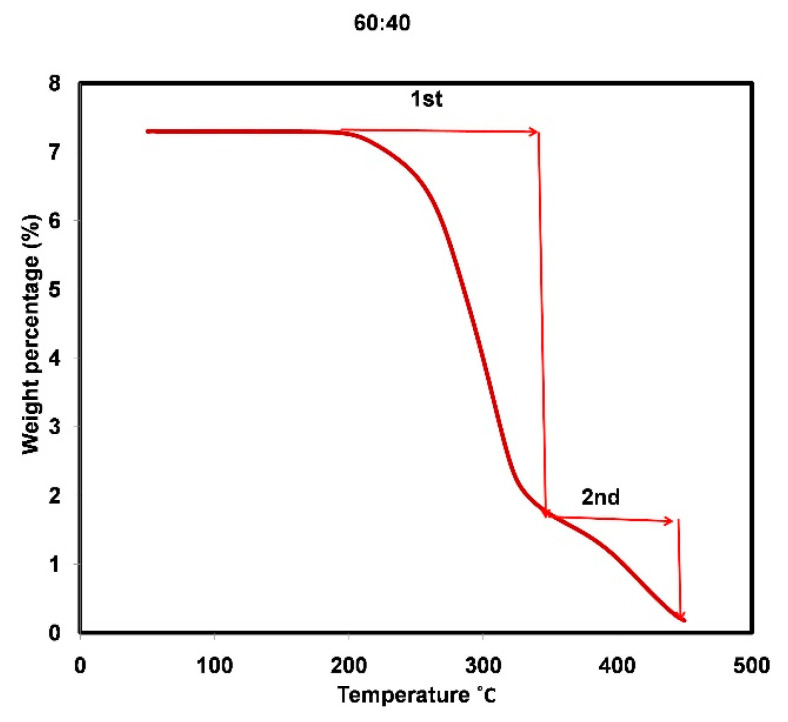
TGA analysis of the solvent-treated microsphere-templated monolith prepared at 60:40 template/monomer ratio at temperatures ranging from 50 to 700 °C.

The thermal stability of the resulting solvent-treated microsphere-templated monoliths was further analyzed. Thermogravimetric analysis (TGA) determines the thermal stability of the material, indicating its stability against elevated temperature [22], as presented in Figure 7. The first deterioration of the monolith prepared using a 60:40 template/monolith ratio occurred at 243 °C, with the second degradation beginning at 332 °C. At 700 °C, the monolith samples had been completely degraded and converted into ashes. A previous study reported monolith degradation at a lower temperature of around 200 °C. That monolith was made of similar monomer chemicals, but was templated using porogen as prepared by Acquah, Danquah, Moy, Anwar and Ongkudon [22]. Yusuf, et al. [34] also reported initial monolith degradation at 210 °C in their study. These findings suggest that the thermal stability of the microsphere-templated monolith was slightly enhanced.

### 3.5. Filtration Ability of Microsphere-Templated Porous Monoliths

**Figure 8:**
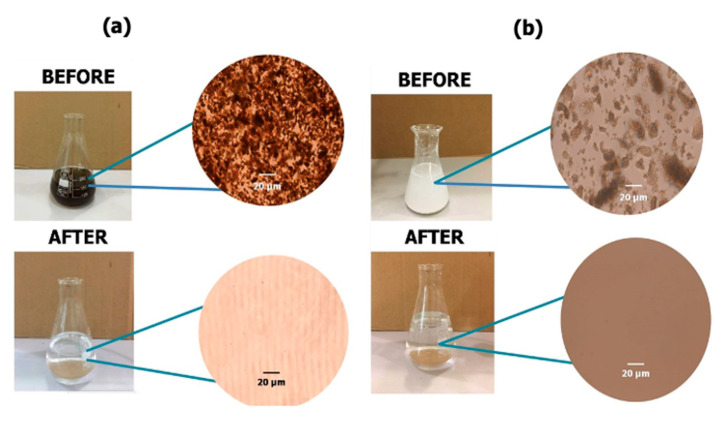
Microscopic images of (**a**) animal pond wastewater and (**b**) laboratory wastewater before and after the filtration process using the developed microsphere-templated porous monolith.

Monoliths are employed for the removal of water-soluble dyes, heavy metal ions, and emulsified oils from water, and demonstrate a high separation efficiency [35,36]. In this study, the efficiency of the microsphere-templated porous polymethacrylate monoliths for removing particulate matter present in wastewater samples was evaluated by microscopic observation and the analysis of the pH value, turbidity and total suspended solids (TSS) of the samples (before and after treatment process). Figure 8 shows the distinguished optical and microscopic images of the wastewater samples before and after filtration. Before the filtration process, the presence of particulate matter can be clearly observed in both samples. The resultant filtered water samples became clear solutions following the filtration process.

Both samples demonstrated an initial pH of 8.8 (Table 2). The turbidity and TSS of the animal pond wastewater were determined to be 80.7 mg/L and 52 mg/L, respectively. Meanwhile, the laboratory wastewater demonstrated turbidity of more than 1000 mg/L and TSS of 662 mg/L, indicating the presence of more particulate matter compared to the animal pond wastewater. According to Oliveira, et al. [37], the TSS value indicates the quality of the sample by indicating the presence of particulate matter in the water. Similar to TSS, turbidity represents the cloudiness of the water due to the presence of different types of particulates such as organic matter, clays, or silts, depending on the origin of samples [38].

**Figure 9:**
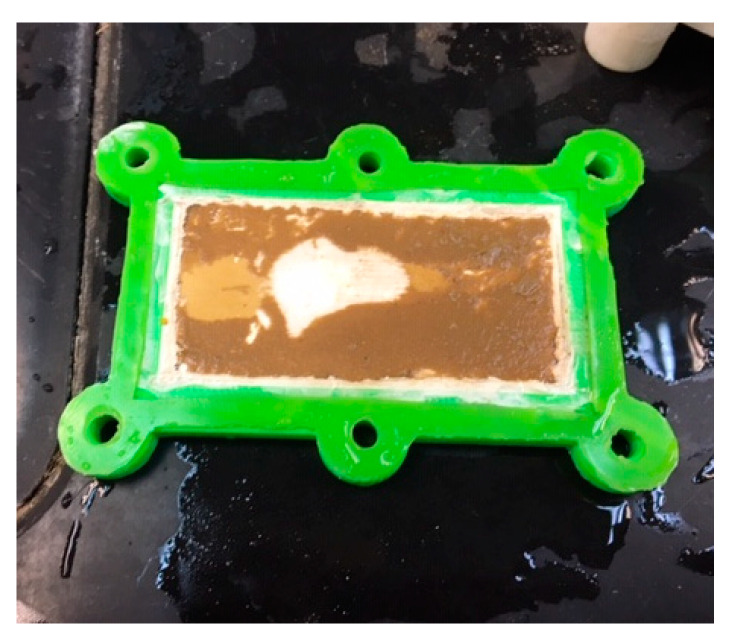
Waste residues on monolith surface upon completion of wastewater filtration.

The values of turbidity and TSS were reduced significantly, to 2.69 mg/L and 1.60 mg/L, respectively, as observed in the animal pond wastewater after the filtration. More interestingly, the laboratory wastewater, which presented a very high concentration of turbidity and TSS in initial testing, exhibited a drastic reduction to almost negligible values of turbidity and TSS (below 1.0 mg/L). The monolith demonstrated efficient filtration performance, successfully eliminating about 96.4–96.9% of the turbidity and TSS in the animal pond wastewater and 99.9% in the laboratory wastewater. The levels of turbidity and TSS are within the Class I category, which denotes water bodies with excellent quality based on the Interim National Water Quality Standard [39]. It is important to note that the laboratory-scale fabricated monolith was able to filter up to 3 L of wastewater in one cycle of the filtration process. The waste residues were remained on the surface of the monolith, as shown in Figure 9, and can be cleaned off by rinsing the monolith with water, enabling subsequent usage of the monolith.

The results of this study collectively demonstrate the suitability of the microsphere-templated monoliths for wastewater treatment. In future study, the efficiency of the monoliths for the removal of particulate matter can be tested on various types of wastewater, including residential and industrial effluents. Furthermore, their ability to remove other contaminants such as biochemical oxygen demand (BOD), chemical oxygen demand (COD), nitrate, phosphorus, and other organic compounds can be further evaluated.

## 4. Conclusions

The results presented in this study reveal that porous monoliths synthesized using polymeric microspheres as a template for pore development can be applied in wastewater treatment applications. A template/monomer ratio of 60:40 were demonstrated to be the best conditions for monolith fabrication, resulting in homogeneous pore distributions at all monolith cross-sections (inner, middle, and outer). Solvent treatment was found to be superior to thermal treatment for the removal of microsphere templates from monolith structures. The fabrication procedure successfully produced a monolith that was scalable and thermally stable at temperatures up to 243 °C. Furthermore, the newly fabricated monoliths were demonstrated to be efficient for the removal of particulate matter present in wastewater. The water quality analyses of the tested animal pond and laboratory wastewaters demonstrated the removal of up to 96–99% of TSS and turbidity. It can be concluded that the pores generated across the monolith structures are able to separate larger and smaller molecules in mixture solutions, which is beneficial for water filtration applications, biomolecule separation, and chromatographic systems.

## Figures and Tables

**Table 1:** Pore size analysis of micro emulsion-based polystyrene microsphere templates prepared at different polymer concentrations.

Polymer Concentration (wt%)	Average Diameter (μm)	Polydispersity Index (PDI)
10	0.9	0.537
20	2.4	0.932
30	2.7	0.999
40	3.4	0.917

**Table 2:** Water analysis of wastewater before and after filtration using monoliths prepared using template-directed porous monolith.

Water Quality	Animal Pond Wastewater		Laboratory Wastewater	
	before	after	before	after
Color	Dark brown	Clear	White	Clear
Presence of particulate matter	Yes	No	Yes	No
pH	8.8	8.9	8.8	8.9
Turbidity (mg/L)	80.7	2.69	>1000	0.91
Total suspended solid (mg/L)	52.00	1.60	662.0	0.50

## Data Availability

Data availabilities are from the authors.

## Supplementary Materials

The following are available online at https://www.mdpi.com/article/10.3390/ma14237165/s1, Figure S1: Images of monolith outer part at 50:50 template/monomer ratio. (a) Original SEM image, (b) threshold of the image set, and (c) the particle outline created with Image J software for the particle size measurement. Figure S2: Images of monolith middle part at 50:50 template/monomer ratio. (a) Original SEM image, (b) threshold of the image set, and (c) the particle outline created with Image J software for the particle size measurement. Figure S3: Images of monolith inner part at 50:50 template/monomer ratio. (a) Original SEM image, (b) threshold of the image set, and (c) the particle outline created with Image J software for the particle size measurement. Figure S4: Images of monolith outer part at 60:40 template/monomer ratio. (a) Original SEM image, (b) threshold of the image set, and (c) the particle outline created with Image J software for the particle size measurement. Figure S5: Images of monolith middle part at 60:40 template/monomer ratio. (a) Original SEM image, (b) threshold of the image set, and (c) the particle outline created with Image J software for the particle size measurement. Figure S6: Images of monolith inner part at 60:40 template/monomer ratio. (a) Original SEM image, (b) threshold of the image set, and (c) the particle outline created with Image J software for the particle size measurement. Figure S7: Images of monolith outer part at 70:30 template/monomer ratio. (a) Original SEM image, (b) threshold of the image set, and (c) the particle outline created with Image J software for the particle size measurement. Figure S8: Images of monolith middle part at 70:30 template/monomer ratio. (a) Original SEM image, (b) threshold of the image set, and (c) the particle outline created with Image J software for the particle size measurement. Figure S9: Images of monolith inner part at 70:30 template/monomer ratio. (a) Original SEM image, (b) threshold of the image set, and (c) the particle outline created with Image J software for the particle size measurement.
