# Supplementary material for: Preparation of Polystyrene Microsphere-Templated Porous Monolith for Wastewater Filtration
Source: Materials (Basel). 2021 Nov 25;14(23):7165. doi: 10.3390/ma14237165 (PMC8658112; doi:10.3390/ma14237165)
Supplement: Supplementary file 1 [file materials-14-07165-s001.zip › materials-1446394-supplementary.pdf]

## SUPLLEMENTARY MATERIALS

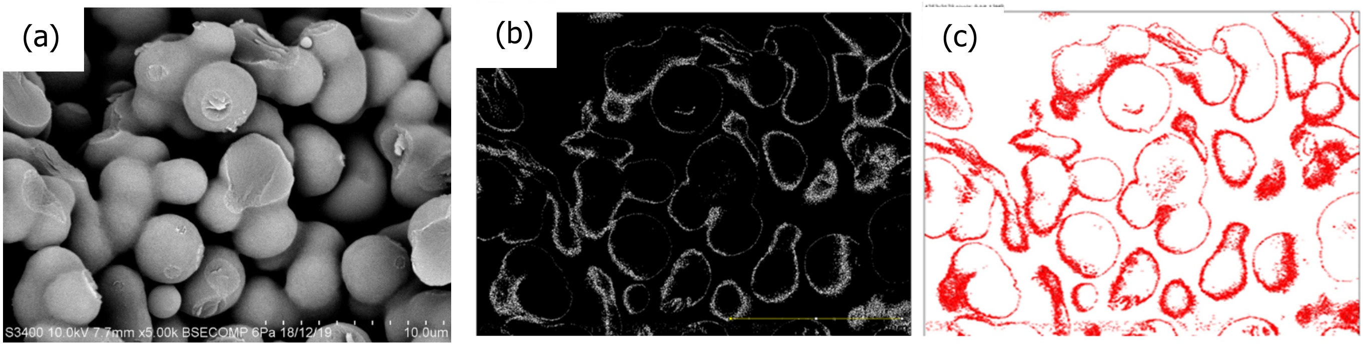


**Figure S1.** Images of monolith outer part at 50:50 template/monomer ratio. (a) Original SEM image, (b) threshold of the image was set, and (c) the particle outline created with Image J software for the particle size measurement.

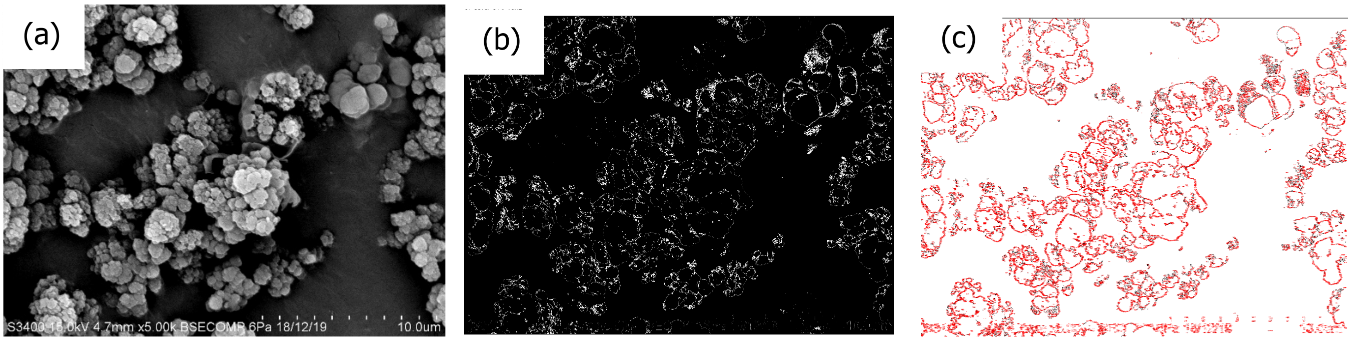


**Figure S2.** Same as Figure S1 for middle part at 50:50 template/monomer ratio.

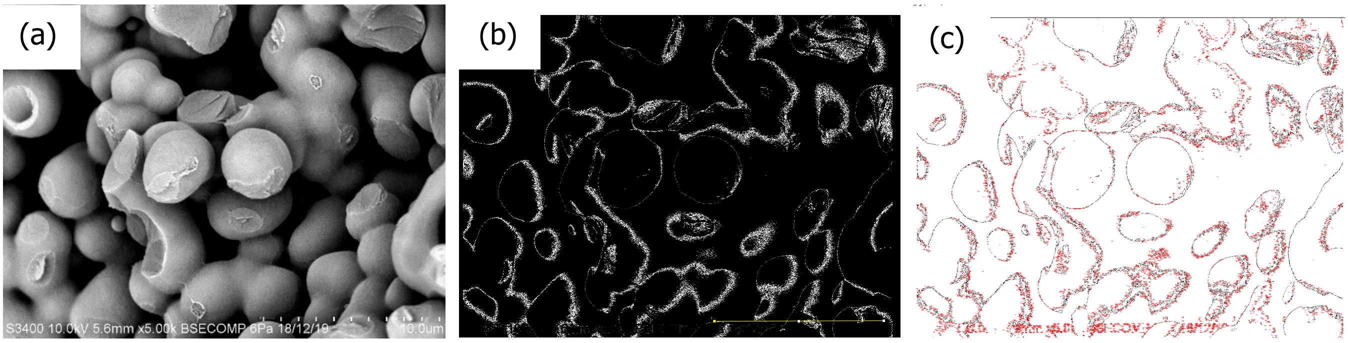


**Figure S3.** Same as Figure S1 for inner part at 50:50 template/monomer ratio.

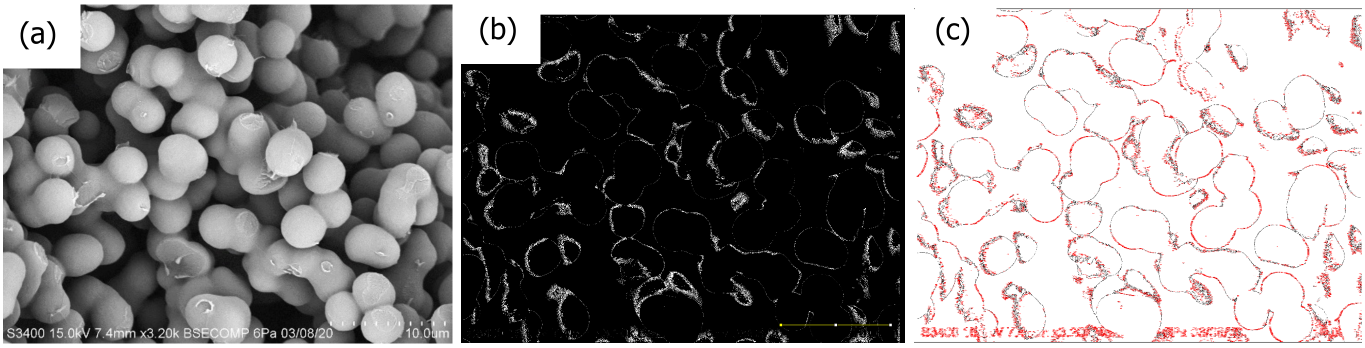

**Figure S4.** Same as Figure S1 for outer part at 60:40 template/monomer ratio.

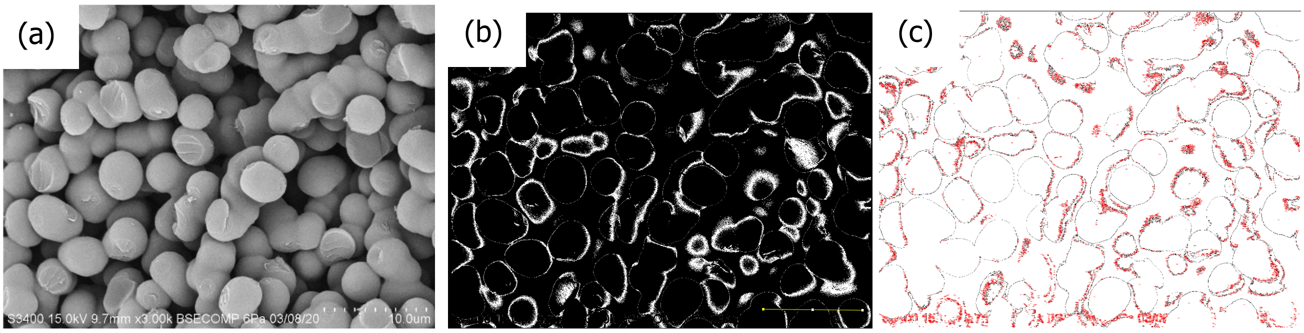


**Figure S5.** Same as Figure S1 for middle part at 60:40 template/monomer ratio.

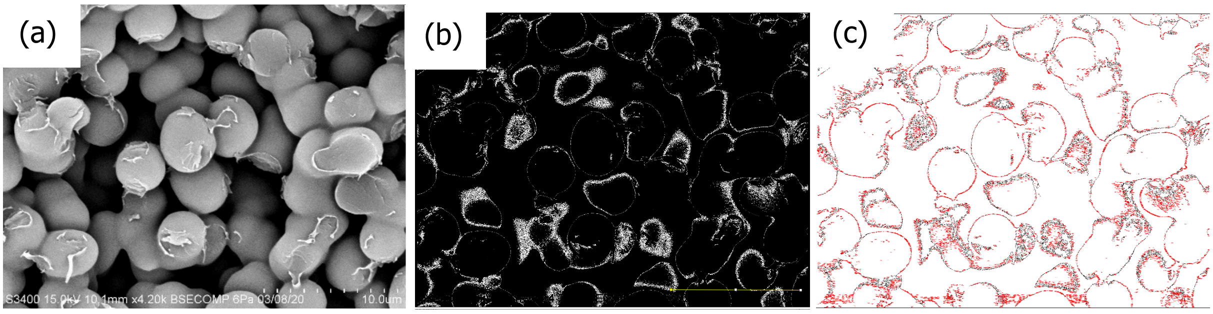


**Figure S6.** Same as Figure S1 for inner part at 60:40 template/monomer ratio.

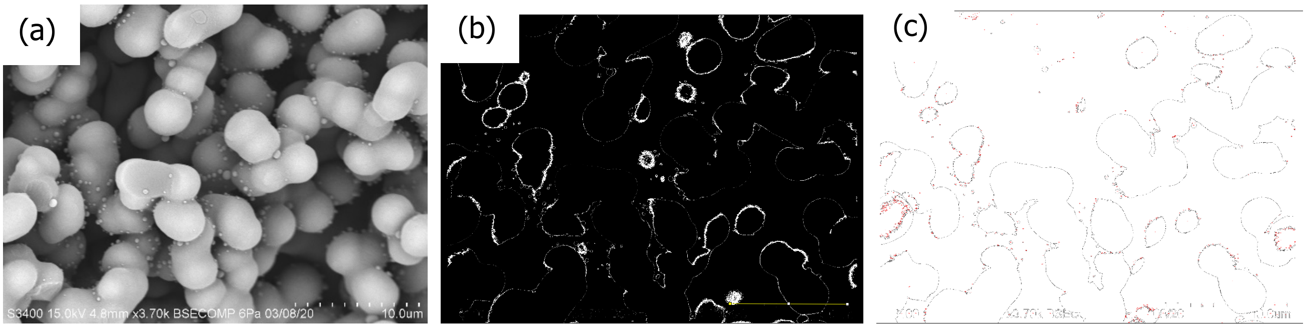


**Figure S7.** Same as Figure S1 for outer part at 70:30 template/monomer ratio.

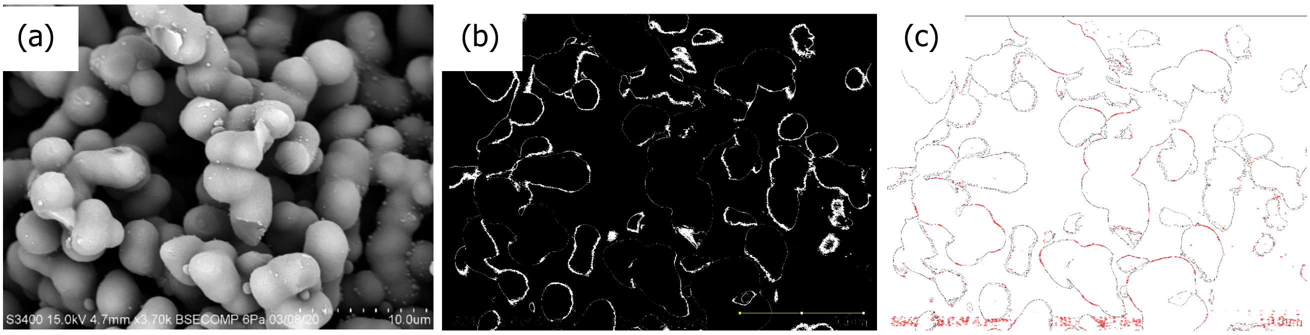


**Figure S8.** Same as Figure S1 for middle part at 70:30 template/monomer ratio.

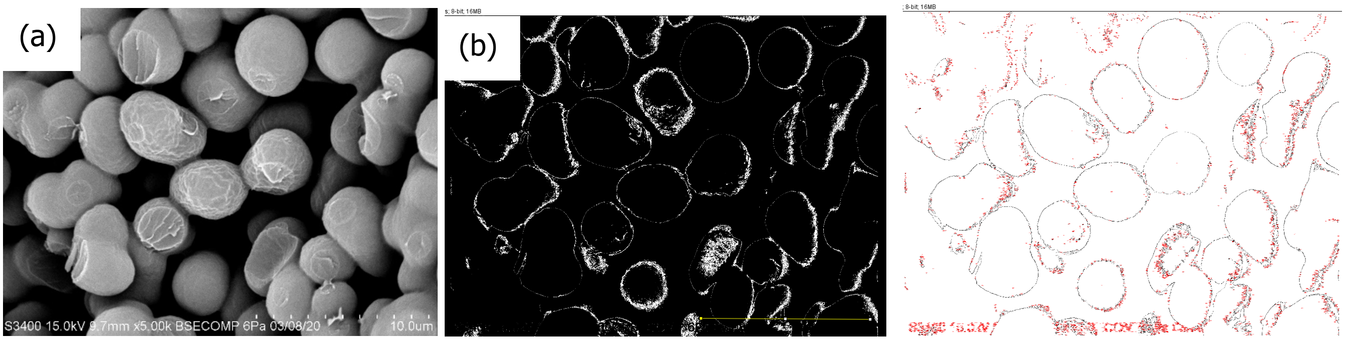


**Figure S9.** Same as Figure S1 for inner part at 70:30 template/monomer ratio.
